# The relationship between perceived parental rearing behaviors and school adjustment of adolescent cancer survivors in Korea: A cross-sectional study

**DOI:** 10.1097/MD.0000000000007758

**Published:** 2017-08-11

**Authors:** Sunhee Lee, Dong Hee Kim

**Affiliations:** a College of Nursing, Catholic University, Seocho-gu; b College of Nursing, Sungshin University, Dobong-ro, Kangbuk-gu, Seoul, Korea.

**Keywords:** cancer, parental rearing behavior, school adjustment

## Abstract

Return and adjustment to school in adolescents who have survived cancer have become of increasing interest as the numbers of childhood cancers survivors have grown due to advances in treatments. Perceived parental rearing behavior is an important factor related to school adjustment. This study examined the relationships between maternal and parental rearing practices, general characteristics, and school adjustment in adolescent cancer survivors in Korea. We conducted a descriptive, exploratory study of 84 adolescents with cancer using the Korean version of the Fragebogen zum erinnerten elterlichen Erziehungsverhalten: FEE (Recalled Parental Rearing Behavior) and a school adjustment measurement. Descriptive, Pearson correlational, and multiple regression analyses were used to investigate the data. In bivariate analysis, age (*r* = −0.358, *P* < .05), mother's emotional warmth (*r* = 0.549, *P* < .01), and father's emotional warmth (*r* = 0.391, *P* < .05) were significantly associated with school adjustment. However, the results of multiple regression analysis showed that only mother's emotional warmth (β = .720, *P* < .05) was significantly associated with school adjustment. Adolescent cancer survivors who reported higher mother's emotional warmth exhibited better school adjustment. This finding indicates that it is important to help parents of adolescent cancer survivors enhance their parental rearing behaviors, such as emotional warmth, to help adolescents adjust to school.

## 1 Introduction

As a result of improvements in treatment for childhood cancer, the number of survivors of this illness has been increasing worldwide.^[1,2]^ Thus, researchers have focused on the normalcy and social reintegration of children who survive cancer. Child cancer survivors are now expected to return to school and to adjust well, either while obtaining treatment or shortly after completing treatment. Returning to school after cancer treatment may bring hope that the child can also return to regular development.

School is an important factor in an adolescent's life.^[3]^ Several decades of research have demonstrated that adolescents’ experiences at school and adjustment to school can exert both positive and negative influences on their development. However, adolescence itself is a known period of decline with respect to numerous school-related outcomes. Particularly, adolescent cancer survivors are vulnerable to adjustment difficulties and to experiencing their school contexts as socially and academically alienating.^[4]^ Furthermore, many adolescent cancer survivors experience residual physical, behavioral, emotional, or social sequelae associated with the disease or its treatment, and these problems can make it difficult for them to adjust to school.^[5]^

To improve school adjustment and normalize the lives of adolescent cancer survivors, it is important to identify the factors related to their school adjustment. This knowledge will provide direction for the development of concrete interventions to foster school adjustment. However, we currently lack information about factors related to adolescent cancer survivors’ school adjustment.

School adjustment is affected by various individual, family, and school factors.^[6–8]^ Parental rearing behavior has been suggested to play a particularly important role in adolescent development.^[9,10]^ Adolescence is a time of increased peer interactions, although the parent–child relationship traditionally remains strong throughout life in Korea.^[11]^ Adolescents with chronic conditions like cancer may be especially close to their parents because parents help them to manage their condition and medications. Therefore, parental rearing behaviors may have strong effects on adolescent cancer survivors.

Parental rearing behavior is a multidimensional concept, so an effective approach would consider specific domains of parental rearing behaviors. Although fathers have been less involved in managing children's chronic illnesses than mothers in Korean culture, fathers’ importance in the family has become clearly recognized, and they are no longer relegated to the sidelines.^[12]^ Thus, we include 3 dimensions of parental rearing behaviors of both the mother and the father in the analyses.

Based on forgoing, this study examines the relationship of both perceived maternal and paternal rearing behaviors included 3 dimensions – emotional warmth, rejection/punishment, and control/overprotection – and school adjustment of adolescent cancer survivors in Korea, using a cross-sectional design.

The following hypotheses will be tested:

Hypothesis 1: The general characteristics of participants will be related to school adjustment in adolescent cancer survivors in Korea.
Hypothesis 2: Three dimensions of maternal rearing practices as perceived by adolescents will be related to school adjustment in adolescent cancer survivors in Korea.
Hypothesis 3: Three dimensions of paternal rearing practices as perceived by adolescents will be related to school adjustment in adolescent cancer survivors in Korea.

## 2 Methods

### 2.1 Setting and participants

Our target sample included adolescents 13 to 19 years old who were enrolled in the pediatric oncology department of a university-affiliated tertiary medical center located in the metropolitan Seoul area; had been diagnosed with childhood cancer, seen for regular check-ups after completing cancer treatment, and were considered to be in remission; were attending regular schools; had no health-related complaints at the most recent visit; and had parents who were willing to participate. All patients who met the inclusion criteria were invited to participate in the study, yielding a convenience sample. Participants were calculated as 77 using G∗Power 3.1 sample calculation program^[13]^ with significance level of 0.05, power of 80%, medium effect size of 0.15 for multiple regression, and 3 independent variables. Given the probability of an about 10% loss of samples and for a higher accuracy, at least 90 participants were being considered at first.

Of the initial cross-sectional sample of 94 participants recruited for the study, 90 adolescents want to participate completed the study. After excluding 6 questionnaires due to missing data, we included reports from 84 adolescents without missing data in the final analysis.

### 2.2 Data collection procedure

After obtaining approval from the Severance Hospital Institutional Review Board (4-2010-0579), data were collected with the cooperation of the hospital's pediatric oncology outpatient clinic staff from July 2013 to December 2013. Informed written consent was obtained from the adolescents with parental agreement before their inclusion in the study. The self-report survey questionnaire was given to the adolescents to fill out while they were waiting to be examined for their regular check-ups with the physicians in the outpatient clinic. The typical time to complete the questionnaire was 15 to 20 minutes. Responses to demographic questions were validated by the accompanying adults; the patient's official cancer diagnosis and time since diagnosis were obtained from medical records. At the completion of the questionnaire, participants were given a token of appreciation.

### 2.3 Measurements

#### 2.3.1 Recalled parental rearing behavior

The Fragebogen zum erinnerten elterlichen Erziehungsverhalten: FEE (Recalled Parental Rearing Behavior) questionnaire is the German short version of the Egna Minnen Berträffande Uppfostran (EMBU) for measuring recalled parental rearing behavior.^[14]^ The EMBU examines 3 domains: rejection/punishment, emotional warmth, and control/overprotection of both the mother and father.^[15]^

The FEE was used to measure main independent variable-perceived parental rearing behaviors of adolescents with leukemia. Like the EMBU, it also has 3 subscales that assess rejection/punishment, emotional warmth, and control/overprotection.^[14]^ This self-report instrument consists of 24 items (8 in each subscale) to be answered separately for the mother and father, with responses indicated on a 4-point Likert scale. The subscale scores range from 8 to 32. A higher score on the rejection/punishment scale indicates a higher level of recollection of rejection and punishment in parental rearing behavior. Similarly, higher scores on the emotional warmth and control/overprotection scales indicate greater emotional warmth and recollection of more overprotective parental rearing, respectively.^[14]^ After initial translation into Korean from German by a bilingual assistant, a 2nd bilingual assistant verified the meaning of each sentence using reverse translation.

Regarding the reliability of the instrument, in an earlier study, Cronbach α values were 0.89, 0.86, and 0.74 for the father and 0.87, 0.86, and 0.72 for the mother for rejection/punishment, emotional warmth, and control/overprotection, respectively.^[16]^ In the current study, Cronbach α values for the father were 0.90, 0.77, and 0.70, and those for the mother were 0.91, 0.78, and 0.69, respectively.

#### 2.3.2 School adjustment

School adjustment as a dependent variable was measured using a scale developed by Kim.^[17]^ It comprises 41 items forming 5 subscales that describe adjustment to school environment, teachers, classes, friends, and school life. This self-report instrument uses a 5-point Likert scale (0: strongly disagree, 4: strongly agree), and the scores range from 41 to 205. Cronbach α in this study was 0.92.

#### 2.3.3 General characteristics

Participant age, gender, level of schooling, religion, perceived economic status, and parent level of education were assessed. Diagnosis and time since diagnosis were also assessed as health-related characteristics.

### 2.4 Data analysis

We performed data analysis using PASW software (version 20.0). The percentages, means, and standard deviations (SDs) were calculated for the adolescents’ general characteristics. To capture variations in parental rearing behavior and school adjustment scores, we obtained the means, SDs, and possible ranges of scores. Pearson correlation coefficients were computed to identify the relationships between school adjustment and the other variables, including parental rearing behavior and general characteristics. We conducted multiple regression analysis to identify variables significantly related to the school adjustment of adolescent cancer survivors. Two-tailed *P* < .05 was defined as statistically significant.

## 3 Results

### 3.1 General characteristics of participants and variation in school adjustment according to general characteristics

The percentages of male and female children were 66.7% and 33.3%, respectively. The mean age of the children was 15.55 years old. Overall, 57% of participants were high school students, and 42% were middle school students. Generally, both parents had completed high school. The majority perceived their family's economic status to be middle class (59.5%). About 30% of the participants suffered from leukemia, about 17% from brain tumors, and about 14% suffered from lymphoma. The duration of illness ranged from 6 months to 10 years, with a mean of 2.49 years (Table 1).

**Table 1:**
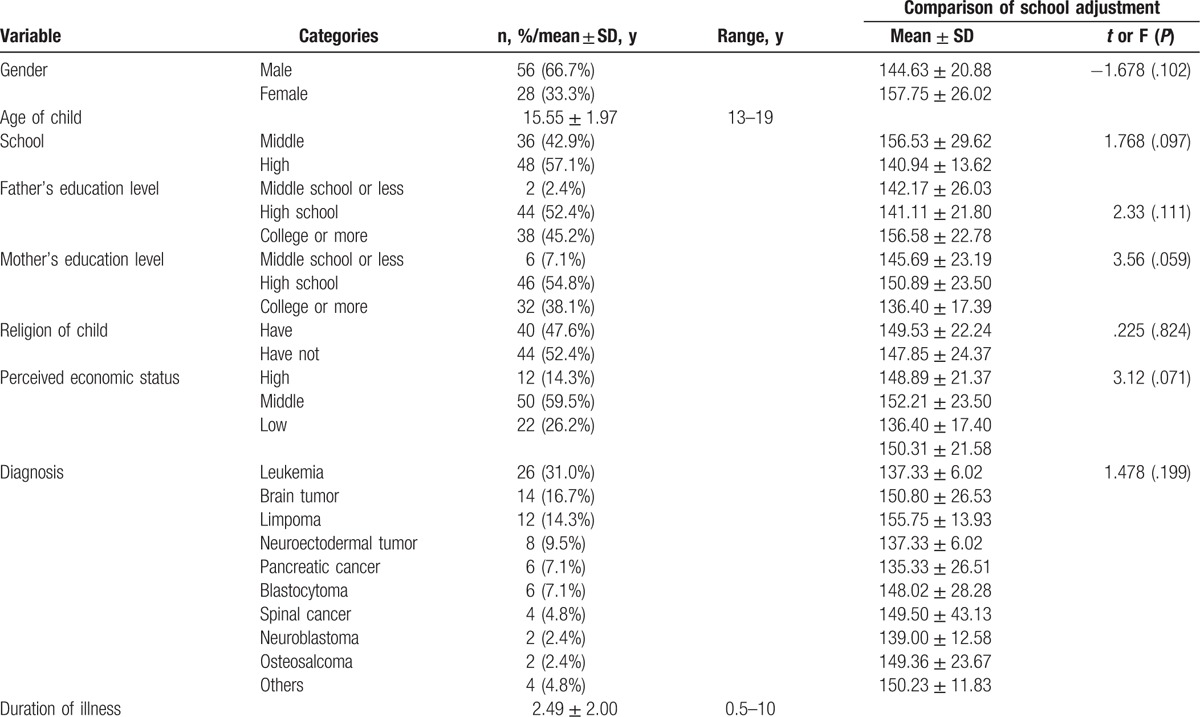
Demographic characteristics of participants (N = 84).

Gender, schooling, parent education level, religion, perceived economic status, and diagnosis did not show statistically significant differences related to school adjustment (Table 1).

### 3.2 Parental rearing behavior and school adjustment scores

The mean scores of perceived mother's emotional warmth, rejection/punishment, and control/overprotection were 22.09 (SD = 5.31; range, 11–32), 12.69 (SD = 3.85; range, 8–24), and 16.67 (SD = 2.77, range, 11–23), respectively. The mean scores of perceived father's emotional warmth, rejection/punishment, and control/overprotection were 21.73 (SD = 5.96, range, 14–32), 10.73 (SD = 3.43, range, 7–21), and 15.95 (SD = 3.18, range, 10–23), respectively. The total school adjustment scores ranged from 96 to 202, with a mean of 145.67 (SD = 23.07).

### 3.3 Variables related to school adjustment

According to Kolmogorov–Smirnov normality test (*P* = .200), school adjustment (main variable) is normally distributed. The relationships between school adjustment and age (*r* = −.358, *P* < .05), mother's emotional warmth (*r* = 0.549, *P* < .01), and father's emotional warmth (*r* = 0.391, *P* < .05) were statistically significant. However, parental rejection/punishment and control/overprotection did not have significant relationships with school adjustment in bivariate analysis (Table 2).

**Table 2:**
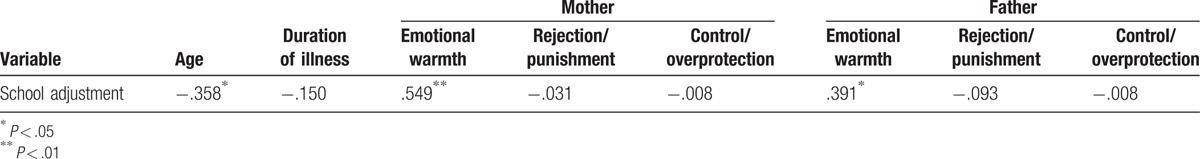
Correlation the variables and resilience (N = 84).

Multivariate analysis assessed the independent variation of the factors explaining school adjustment. The variables included in the regression model were age, mother's emotional warmth, and father's emotional warmth. The only variable significantly associated with school adjustment was mother's emotional warmth (β = .720, *P* < .05), and this variable in the regression model explained 29.0% of the school adjustment in this study (Table 3).

**Table 3:**
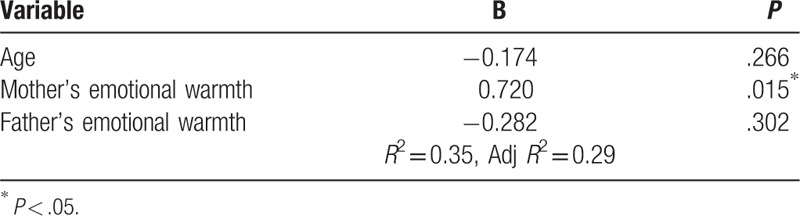
Factors related to school adjustment (N = 84).

## 4 Discussion

Return and adjustment to school after diagnosis of cancer is a challenge and an important issue for adolescent cancer survivors. In this study, exploring the school adjustment of adolescent cancer survivors, factors related to school adjustment and parenting positively affected school adjustment of adolescent cancer survivors.

The mean school adjustment score of the adolescent survivors of cancer in this study was 145.67, which is higher than that among healthy Korean adolescents. The mean scores of Korean healthy adolescents in other studies using the same school adjustment scale as in this study were 135.30^[18]^ and 129.48.^[19]^ Consistent with this study, adolescent survivors of cancer in those studies achieved higher levels of school and professional education compared to age-matched samples from the general population,^[20]^ and resilience did not differ between adolescent survivors of brain tumors and healthy adolescents.^[21]^ Zuzak et al^[22]^ found that pediatric cancer survivors rated their health-related quality of life as normal or higher than healthy controls, and only 4 of 21 patients (19%) had difficulty making friends. Phillips and Jones^[23]^ stated in their qualitative study that adolescent survivors of childhood cancer reported the desire to maintain a positive focus on school. Many adolescent survivors maintain a positive attitude in order to endure the cancer experiences.^[23]^ These positive attitudes probably reflect higher levels of school adjustment. Therefore, it might be an effective intervention to focus on their positive attitude to school. However, the uncertainty of recurrence and return to school after a long treatment duration must be considered in these students. Therefore, continuous attention and further evaluation regarding school adjustment of cancer survivors are needed.

Our 1st hypothesis about general characteristics of participants was supported by only age. The age is negatively correlated with school adjustment. Older Korean adolescent survivors of cancer are less capable of adjusting to the return to school. The majority of Korean parents hope that their children will enter a prestigious university, with the aims of education of middle and high school in Korea being college preparation rather than development of self-identity.^[24]^ Korean adolescents experience increasing stress regarding academic performance as they advance in school. In addition, Korean adolescents with chronic disease have difficulties catching up with their studies after returning from long hospitalization.^[25]^ A program to help adolescents attending middle or high school readjust to school life after a long absence due to illness is needed. Such a program should consist of sessions for the students as well as for their parents.

Findings from this study partially support our 2nd and 3rd hypothesis. Our findings indicate that the school adjustment of adolescent cancer survivors is significantly correlated with perceived emotional warmth of the parents. Simons-Morton and Crump^[26]^ found that parental warmth was a better predictor of school adjustment than other measures of parenting behaviors. McCoy et al^[27]^ found that school adjustment was influenced by mother's warmth. Consistent with this study, mother's and father's emotional warmth was negatively correlated with adolescent problems such as eating disorder, psychiatric disorder,^[28]^ and gambling disorder.^[29]^ Warmth and stable parenting help adolescent with self-confidence, sociability, and good psychological health. They also may act as protective factors against behavioral problems, therefore positively associating the student's readjustment to school life.^[30]^

Some research has suggested that emotional warmth is influenced by parenting stress. Many parents have reported parenting stress because of the risk of cancer recurrence, even when treatment had been successful.^[31]^ One study of mothers of adolescent cancer survivors found that maternal stressors and worries were largely related to fears of cancer recurrence, the aftereffects of treatment, and concerns regarding psychosocial difficulties.^[32]^ Vrijmoet-Wiersma et al^[33]^ stated that, 5 years after treatment, 40% of the mothers continued to report heightened stress levels compared with the reference group. This parenting stress of mothers of adolescent cancer survivors is negatively correlated with emotional warmth in parental rearing.^[34]^ Therefore, it is important to develop programs to help parents of adolescent cancer survivors to improve their emotional warmth in the face of parenting stress.

Furthermore, in multivariate regression analysis in the present study, perceived mother's emotional warmth was the single most important factor found to associate with school adjustment, while perceived father's emotional warmth did not associate with school adjustment. Traditional Korean fathers’ roles are based in Confucianism. Korean fathers are the breadwinner for the family and typically are strict and patriarchal,^[35]^ similar to other Asian countries such as China and Japan, whereas mothers are in charge of raising the children. Korean adolescents’ behaviors thus tend to be influenced more by the mother than the father. Recently, fathers’ participation in parenting has steadily increased, and the roles of fathers have changed. Subsequently, studies about the parenting style of fathers have been performed.^[36]^ Paternal parenting has a profound positive impact on a child's life.^[37]^ Therefore, future studies of the role of fathers in children and adolescents survivors of cancer are needed.

Our study has several limitations. Since participants were recruited from a single medical center, the generalizability of this study is likely limited. In addition, school adjustment of adolescent survivors cancer is related to numerous variables such as self-esteem and academic performance in addition to the variables considered in this study. And there is a potential bias from unmeasured confounders which are ubiquitous problems for most observational studies. Therefore, more studies with a more representative sample and considering other important variables are recommended. Finally, this study is a cross-sectional design, which has recall bias and may result in different findings from a longitudinal study. A longitudinal study to identify a variety of features and causality of school adjustment and related factors would be recommended. Despite the limitations, this study was meaningful in identifying the importance of parenting warmth as a vehicle to enhance the school adjustment of adolescent cancer survivors.

## 5 Conclusion

This study sought to identify the relationships between maternal and paternal parental rearing practices, general characteristics, and school adjustment in adolescent cancer survivors in Korea. Older age of adolescent cancer survivors was associated with difficulty adjusting to the school environment. Therefore, an intervention program that can improve school adjustment for adolescent cancer survivors would be useful.

Both maternal and paternal emotional warmth significantly improved cancer survivors’ adjustment to school. The parent–child bond remains strong throughout adolescence in Korea; this is especially true for adolescent cancer survivors and their parents because they spend more time together due to the child's condition. Intervention strategies that enhance positive parenting of adolescent cancer survivors would contribute to improving school adjustment.

Furthermore, adolescent cancer survivors who reported higher maternal emotional warmth showed better school adjustment than their counterparts. This suggests that mothers still play a bigger parenting role in Korea than fathers. Therefore, the most effective intervention might be an educational program focusing on maternal positive parenting.
